# ASAS-NANP symposium: Mathematical Modeling in Animal Nutrition: The power of identifiability analysis for dynamic modeling in animal science:a practitioner approach

**DOI:** 10.1093/jas/skad320

**Published:** 2023-11-22

**Authors:** Rafael Muñoz-Tamayo, Luis O Tedeschi

**Affiliations:** Université Paris-Saclay, INRAE, AgroParisTech, UMR Modélisation Systémique Appliquée aux Ruminants, 91120 Palaiseau, France; Department of Animal Science, Texas A&M University, College Station, TX 77843-2471, USA

**Keywords:** dynamic modeling, model calibration, parameter estimation, parameter identification, practical identifiability, structural identifiability

## Abstract

Constructing dynamic mathematical models of biological systems requires estimating unknown parameters from available experimental data, usually using a statistical fitting procedure. This procedure is usually called parameter identification, parameter estimation, model fitting, or model calibration. In animal science, parameter identification is often performed without analytic considerations on the possibility of determining unique values of the model parameters. These analytical studies are related to the mathematical property of structural identifiability, which refers to the theoretical ability to recover unique values of the model parameters from the measures defined in an experimental setup and use the model structure as the sole basis. The structural identifiability analysis is a powerful tool for model construction because it informs whether the parameter identification problem is well-posed (i.e., the problem has a unique solution). Structural identifiability analysis is helpful to determine which actions (e.g., model reparameterization, choice of new data measurements, and change of the model structure) are needed to render the model parameters identifiable (when possible). The mathematical technicalities associated with structural identifiability analysis are very sophisticated. However, the development of dedicated, freely available software tools enables the application of identifiability analysis without needing to be an expert in mathematics and computer programming. We refer to such a non-expert user as a practitioner for hands-on purposes. However, a practitioner should be familiar with the model construction and software implementation process. In this paper, we propose to adopt a practitioner approach that takes advantage of available software tools to integrate identifiability analysis in the modeling practice in the animal science field. The application of structural identifiability implies switching our regard of the parameter identification problem as a downstream process (after data collection) to an upstream process (before data collection) where experiment design is applied to guarantee identifiability. This upstream approach will substantially improve the workflow of model construction toward robust and valuable models in animal science. Illustrative examples with different levels of complexity support our work. The source codes of the examples were provided for learning purposes and to promote open science practices.

## Teaser

Identifiability analysis is a powerful, valuable tool for developing robust predictive models. With the help of freely available software, modelers in animal science can easily integrate this analysis into their model developments.

## Introduction

Modeling the dynamics of a biological system is an exercise of translating the knowledge of the phenomena that drives system behavior into ordinary differential equations (**ODE**). Its state variables (sometimes called compartments in animal science literature) and its parameters define a dynamic mathematical model. The parameter values are often unknown and must be estimated from experimental data via parameter identification (also termed parameter estimation, model calibration, or model fitting). Parameter identification is the mathematical process of finding the numerical values of the model parameters that best fit the variables given the available data. Parameter identification is essentially an optimization problem that aims to minimize the distance between the ­model-predicted and observed (measured data) values. The problem can be formulated in the maximum likelihood approach (Walter and Pronzato, 1997) or within a Bayesian framework (Reed et al., 2016). For nonlinear problems, the optimization can result in multiple local solutions. To avoid the convergence of local solutions, global and hybrid global-local optimization methods have been developed (Walter and Pronzato, 1997; Banga, 2008). As modelers, we are interested in knowing whether the optimization problem has a unique solution. Structural identifiability analysis aims to assess the possibility of estimating a unique best value of the model parameters from available measurements. This identifiability property is of particular importance in models where the parameters have biological meaning. The evaluation of structural identifiability is only based on the mathematical structure of the model but does not depend on the actual data. This qualitative property is based on the assumption that the model is accurate (no characterization error), the measurements are noise-free exact (no measurement errors), and that the model inputs and measurement times can be chosen freely without any constraint. Accordingly, with these ideal assumptions, the property of structural identifiability does not depend on the limitations imposed by the quality and quantity of the data available for parameter identification. The rigorous mathematical framework of structural identifiability has been discussed by Walter and Pronzato (1997), while a simple introduction targeted to the animal science community was provided by Muñoz-Tamayo et al. (2018).

This paper aims to illustrate the power of identifiability analysis for developing robust predictive models. We also aimed to promote the use of identifiability analysis within the modeling construction workflow in animal science.

## Defining Parameter Identifiability

### Structural identifiability

Let us consider the following ODE model shown as follows:


$$\begin{array}{l} \frac{dx\left(t\right)}{dt}=f\left(x,u,p\right),\; \end{array}$$

$$\begin{array}{l} y\left(t\right)=g\left(x,u,p\right),\;\;x\left(0\right)=x_{0}\left(p\right) \end{array}$$ (1)

where **x** is the vector of state variables, **y** is the output vector (measurements), **u** is the input vector, and **p** is the parameter vector. The model structure is defined by the vector functions **f**, **g**_,_ which can be linear or nonlinear. A parameter $p_{i}$ is structurally identifiable if it can be uniquely recovered from information on the input and output variables. This property translates mathematically as follows:


$$\begin{array}{l} y\left(t,\hat{p}\right)=y\left(t,p^{*}\right)\Rightarrow {\hat{p}}_{i}=p_{i}^{*} \end{array}$$ (2)

Structural identifiability can be local or global. The parameter $p_{i}$ is structurally locally identifiable if it can be estimated in a neighborhood of its nominal value, but a finite number of possible values exist in the parameter space that holds equation (2). The parameter $p_{i}$ is structurally globally identifiable if it can be uniquely estimated in the whole parameter space (Barreiro and Villaverde, 2022, preprint). If none of the previous conditions hold, the parameter $p_{i}$ is non-identifiable. It should be noted that the assessment of identifiability analysis may, in some cases, depend on the initial conditions of the state variables (Denis-Vidal et al., 2001; Saccomani et al., 2003). Indeed, in some cases, certain initial conditions may lead to the loss of identifiability. Joubert et al. (2021) proposed a method to identify some problematic initial conditions impacting parameter identifiability.

### Existing methods and software tools

A variety of methods exists to test the structural identifiability of dynamic models. They include the Laplace transform (for linear models), direct test, differential algebra, Taylor series, and generating series Chis et al., 2011; Miao et al., 2011). In addition, many software tools are freely available to assess structural identifiability (locally or globally). The outcome given by software tools is qualitative; the software displays which parameters (or combination of parameters) are identifiable and which are not. Table 1 lists the availability of common and recent software tools. These software tools include DAISY (Bellu et al. 2007; Saccomani et al. 2019), ObservabilityTest (Sedoglavic 2002), IdentifiabilityAnalysis (Karlsson et al. 2012), STRIKE-GOLDD 4.0 (Villaverde et al. 2016; Díaz-Seoane et al. 2023; Hong et al. 2019), Structural Identifiability Toolbox (Ilmer et al. 2021), StrucID (Stigter and Joubert 2021), StructuralIdentifiability (Dong et al. 2022, preprint), and NonlinearObservabilityTest (Shi and Chatzis 2022). Some of these tools are implemented in commercial programming languages such as Matlab (https://fr.mathworks.com/products/matlab.html), Mathematica (https://www.wolfram.com/mathematica/), and Maple (https://www.maplesoft.com), while others are implemented in free and open-source environments such as Reduce (https://reduce-algebra.sourceforge.io/), Maxima (https://maxima.sourceforge.io/), and Julia (https://julialang.org/) (Bezanson et al., 2017). Benchmarking studies have been performed to compare identifiability software tools (Raue et al., 2014; Hong et al., 2019; Dong et al., 2022, preprint). Barreiro and Villaverde (2022, preprint) provided a comprehensive benchmarking study assessing 12 software tools for identifiability analysis. Their study discussed the strengths and weaknesses of different tools and provided software selection guidelines. For global identifiability analysis, the authors recommend using the Maple implementation of SIAN and StructuralIdentifability.

**Table 1.** Software tools for structural identifiability analysis

Tool	Description
DAISY	URL: https://daisy.dei.unipd.it Language: Reduce Analysis: local and global solutions Application: rational models Reference: Bellu et al. (2007); Saccomani et al. (2019)
ObservabilityTest	URL: https://github.com/sedoglavic/ObservabilityTest Language: Maple Analysis: local solution Application: rational models Reference: Sedoglavic (2002)
IdentifiabilityAnalysis	URL: https://www.fcc.chalmers.se/software/other-software/identifiabilityanalysis/ Language: Mathematica Analysis: local solution Application: rational models Reference: Karlsson et al. (2012)
STRIKE-GOLDD 4.0	URL: https://github.com/afvillaverde/strike-goldd Language: Matlab Analysis: local solution Application: rational and non-rational models Reference: Villaverde et al. (2016); Díaz-Seoane et al. (2023)
GenSSI 2.0	URL: https://github.com/genssi-developer/GenSSI Language: Matlab Analysis: local and global solutions Application: rational and non-rational models Reference: Chiş et al. (2011); Ligon et al. (2018)
COMBOS	URL: http://biocyb1.cs.ucla.edu/combos/ Language: Maxima, web application Analysis: local and global solutions Application: rational models Reference: Meshkat et al. (2014)
SIAN	URL: https://github.com/alexeyovchinnikov/SIAN-Julia https://github.com/pogudingleb/SIAN Language: Maple and Julia Analysis: local and global solutions Application: rational models Reference: Hong et al. (2019)
Structural Identifiability Toolbox	URL: https://maple.cloud/app/6509768948056064 Language: Maple, web application Analysis: local and global solutions Application: rational models Reference: Ilmer et al. (2021)
StrucID	URL: available upon request from the authors Language: Matlab Analysis: local solution Application: rational and non-rational models Reference: Stigter and Joubert (2021)
StructuralIdentifiability	URL: https://github.com/SciML/StructuralIdentifiability.jl Language: Julia Analysis: local and global solutions Application: rational models Reference: Dong et al. (2022, Preprint)
NonlinearObservabilityTest	URL: https://eng.ox.ac.uk/non-lineardynamics/resources/ Language: Matlab Analysis: local solution Application: rational models Reference: Shi and Chatzis (2022)

### Practical identifiability

Developments in structural identifiability analysis have reached a high degree of maturity, which has led some authors to declare that determining structural identifiability is not longer an issue (Wieland et al., 2021). Since structural identifiability is a qualitative property, a quantitative assessment of the parameters’ accuracy is needed to fully characterize the parameters’ identifiability for a given experimental data set. This assessment is related to the notion of practical identifiability, and it should be said that structural non-identifiability implies practical non-identifiability.

Briefly, practical identifiability analysis is centered on the numerical determination of the confidence intervals of the parameter estimates. Different methods are available for the computation of parameter confidence intervals, including the Fisher information matrix (**FIM**)-based approach, Monte-Carlo simulation, Bayesian method, and profile likelihood. Practical identifiability methods were reviewed by Lam et al. (2022), and their characteristics in terms of computational cost and statistical interpretability were discussed by Villaverde et al. (2022). Software tools for parameter identification allow for practical identifiability analysis based on the FIM (Muñoz-Tamayo et al., 2009; Balsa-Canto et al., 2016), sensitivity analysis (Soetaert and Petzoldt, 2010), or profile likelihood approach (Raue et al., 2015).

It is important to emphasize that the conceptual development of practical identifiability analysis is less mature than structural identifiability (Wieland et al., 2021). The logic sequence between the two identifiability notions of data collection explains that structural identifiability is also called a priori identifiability, while practical identifiability is termed a posteriori identifiability. The joint integration of structural and practical identifiability analyses offers a powerful armory to tackle the parameter identification of models of biological systems (Miao et al., 2011; Saccomani and Thomaseth, 2018).

In addition to structural and practical identifiability analyses, sensitivity analysis can provide helpful information on parameter identifiability. A sensitivity analysis study allows for assessing how the model outputs are affected by different sources of variation, including the model parameters (Saltelli et al., 2000). That is, how the change of a parameter impacts the behavior of the model output. Sensitivity analysis is central to identifying the phenomena that play a significant role in system behavior and ranking the model parameters regarding their influence on the model outputs. Various model developments in animal science include sensitivity analysis to evaluate the effect of variation of parameters and input variables on the model behavior (Doeschl-Wilson et al., 2009; Tedeschi and Fox, 2009; Puillet et al., 2016; Dougherty et al., 2017; van Lingen et al., 2019).

The FIM-based approach for practical identifiability is based on the calculation of the sensitivity of the model to its parameters. Indeed, if the model outputs are highly sensitive to a small perturbation of a given parameter, this parameter is likely to be identifiable (Miao et al., 2011). The information provided by sensitivity analysis is useful, for example, to discard parameters with little influence on the model outputs and to reduce the number of parameters estimated with the model calibration.

### Parameter Identifiability in Animal Science

In the animal nutrition field, the concept of structural identifiability was introduced by Boston et al. (2007) and further detailed by Tedeschi and Boston (2010), focusing on linear models. Later on, Muñoz-Tamayo et al. (2018) expanded on the mathematical elements and notions associated with the structural identifiability analysis for nonlinear ODE models and discussed the relevance of identifiability analysis in the modeling construction. The paper illustrates relevant examples that demonstrate the consequences of ignoring identifiability testing when the identifiability question is relevant. It also shows the implications that can have the use of identifiability on the costs of the experimental design. To emphasize the relevance of identifiability analysis, let us consider a compartmental model (equations (3) and (4)) that is prevalent in animal science, and their applications include, for example, the study of rumen digesta passage (Poppi et al., 2001) and physiological regulations (Puillet et al., 2008).


$$\begin{array}{l} \frac{dx_{1}}{dt}=-p_{1}\cdot x_{1} \end{array}$$ (3)

$$\begin{array}{l} \frac{dx_{2}}{dt}=p_{1}\cdot x_{1}-p_{2}\cdot x_{2} \end{array}$$ (4)

**Figure 1.**
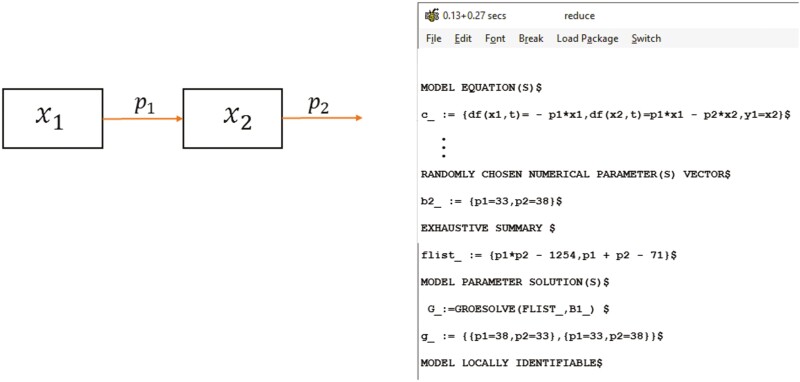
Scheme of a compartmental model and the output of identifiability testing with the DAISY software (Bellu et al. 2007). If only *x*_2_ is measured and the initial condion *x*_1_(0) is unknown the parameters are locally identifiable. Two solutions exist where the values of the parameters are switched between the solutions.

This compartmental model has two parameters $p_{1}$, $p_{2}$ and two state variables $x_{1}$, $x_{2}$. We consider that only $x_{2}$ can be measured, and the initial condition $x_{1}\left(0\right)$ is unknown. Under this condition, the parameters are locally identifiable. The results using DAISY are displayed in Figure 1. For the sake of clarity, it should be said that the algorithm implemented in DAISY uses random numerical values for the parameters to resolve the system of equations of the identifiability test. These random numerical values are those displayed in ­Figure 1. They are not the actual values of the parameters. As previously mentioned, the numerical values of the parameters are obtained from a parameter identification routine that minimizes the distance between the model-predicted and observed values. We observed that DAISY gives two solutions for the identifiability where the values of the parameters are switched between the two solutions. That means that if the true value of the parameters are $p_{1}^{*},\;p_{2}^{*}$, the correct solution is $p_{1}=p_{1}^{*};p_{2}=p_{2}^{*}$, while the second (wrong) solution is $p_{1}=p_{2}^{*};p_{2}=p_{1}^{*}$. This outcome results from the fact that, to solve the parameter estimation problem, the initial condition $x_{1}\left(0\right)$ should also be considered as a parameter to be estimated because its value is unknown. The solutions found by DAISY are associated with two different $x_{1}\left(0\right)$. Let’s assume that the modeler has a perfect data set. The optimization algorithm resolves the wrong parameter values when performing the calibration procedure. The modeler, unaware of the identifiability analysis, could conclude that the parameter estimation is satisfactory because the model fitting is perfect. What are the consequences of this situation? Firstly, the parameters $p_{1}$, $p_{2}$ might have biological meaning, so conclusions based on the specific values of the parameter estimates will be wrong. Note, for example, that $p_{1}$ and $p_{2}$ can have a different order of magnitude, which is a typical case of systems where internal processes occur at different time scales (i.e., slow vs. fast processes). The second consequence is that with the wrong parameter estimates, the model can represent very well the dynamics of $x_{2}$. However, the model will fail to provide accurate predictions of $x_{1}$. Many model developments aim at providing predictions of unobserved variables; therefore, it is essential to estimate accurate parameters. An identifiability analysis can help to fulfill this goal. In the example, to obtain a unique set of parameters, we only need to know the value of the initial condition $x_{1}(0)$. Note that this example is not the worst-case scenario that the modeler can find because the parameters are at least locally identifiable. The most critical situation is when the parameters are non-identifiable. Muñoz-Tamayo et al. (2018) illustrate an example where the wrong choice of the initial condition can render the parameters non-identifiable. The identifiability of the parameters is guaranteed by simply changing the initial conditions. This capability that the modeler has to set the conditions of an experimental protocol to ensure parameter identifiability before data collection refers to the room of maneuver mentioned above.

With the previous example, we briefly showed the relevance of identifiability analysis. Despite our motivation to promote structural identifiability analysis in the modeling arena in animal science, this approach is still seldom applied to the study of dynamic models. Therefore, in our paper, we focus on dynamic models described by ODE. However, identifiability analysis also applies to statistical models. Examples of identifiability analysis of statistical models used in animal genetics have also been addressed (Cantet and Cappa, 2008; Shariati et al., 2009).

Few dynamic modeling studies integrate structural identifiability analysis. These studies include a model for the transmission of mastitis in dairy cows (White et al., 2002), a Gompertz-based model to describe the body weight dynamics of piglets at weaning (Revilla et al., 2019), a model to quantify the response of feed intake of pigs facing a perturbation (Nguyen-Ba et al., 2020), a model to characterize body condition score variations in sheep (Macé et al., 2020, Preprint), a model to describe the methanogenesis by rumen archaea (Muñoz-Tamayo et al., 2019a), and a model to describe in vivo methane production from cattle (Muñoz-Tamayo et al., 2019b).

Although structural identifiability has been rarely applied in the animal science field, considerations of the practical and numerical issues of the model calibration are obliged aspects that modelers face to find an adequate strategy that facilitates the numerical estimation of the model ­parameters. For example, sensitivity analysis was applied to two mathematical models developed to describe the susceptibility of porcine alveolar macrophages to an RNA virus (Doeschl-Wilson et al., 2016). This approach allows for a reduction in the number of parameters identified by fixing the values of a subset of the parameters. Similarly, sensitivity analysis was used to perform a stepwise fitting procedure to estimate the parameters of a model of the bovine estrous cycle (Boer et al., 2017). A two-step parameter identification strategy to limit practical identifiability issues was implemented to identify the parameters of a lactation model that account for perturbations (Ben Abdelkrim et al., 2021). In a modeling development describing the interaction between the growth rate of the developing embryo and the uterine environment in cows, identifiability analysis was performed to guarantee the unicity of the parameters (Shorten et al., 2018). However, no details were provided about how the identifiability analysis was done. We might infer that the authors refer to practical identifiability analysis extracted from the standard error calculation by the Markov chain Monte-Carlo method. The profile likelihood approach (Murphy and Van Der Vaart, 2000; Raue et al., 2009) was applied to assess the practical identifiability of a model developed to describe the effect of diet composition on sheep weight (Vargas-Villamil et al., 2020).

Our objective in this paper follows up on our previous attempts to promote identifiability analysis in the modeling exercise in our field. We will illustrate the power of identifiability analysis using models at different levels of complexity. Following open science practices (Muñoz-Tamayo et al., 2022), the source codes with the implementation of identifiability analysis for all the examples are freely available (Muñoz-Tamayo, 2023).

## The Power of Model Simplification

We consider here the process of protein hydrolysis in the context of cheese ripening. In this hydrolytic process, lactic acid bacteria break down milk proteins (e.g*.,* β-casein) into various peptides, which are further metabolized. The following model describes the hydrolysis of the intact β-casein by the P_I_-type protease of *Lactococcus lactis* in a batch system (Muñoz-Tamayo et al., 2011).


$$\begin{array}{l} \frac{dx}{dt}=-k\cdot E\cdot \frac{x}{K_{m}\cdot (1+(I/k_{I}))+x} \end{array}$$ (5)

where *x* is the concentration of β-casein (mol/L), *E* (mol/L) is the concentration of the protease, *I* (mol/L) is the concentration of the inhibitor compound(s), *k* is the catalytic rate constant (mol *x*/(mol E⋅min)), $K_{m}$ (mol/L) is the affinity substrate constant, and $k_{I}$ is the inhibition constant (mol/L). The model in equation (5) can be categorized as a mechanistic model. It is derived from a mass balance, and its parameters are biologically meaningful (interpretable).

Let us consider that only the concentration β-casein (mol/L) was measured at different sampling times. The concentration of the enzyme *E* is known and constant (*E* = 1). We will then need information about the inhibitor *I* to set up the parameter identification problem. During the hydrolysis, there is a competition between the intact protein and the released peptides for the active sites of the protease. Indeed, the kinetic function in Eq. (5) is called a competition inhibition kinetic rate. We can then consider that the inhibitor is the sum of all peptides released. Accordingly, $I=\;x_{0}-x$, where $x_{0}$ is the initial concentration of β-casein. The initial concentration $x_{0}$ is known ($x_{0}=10$).

**Figure 2.**
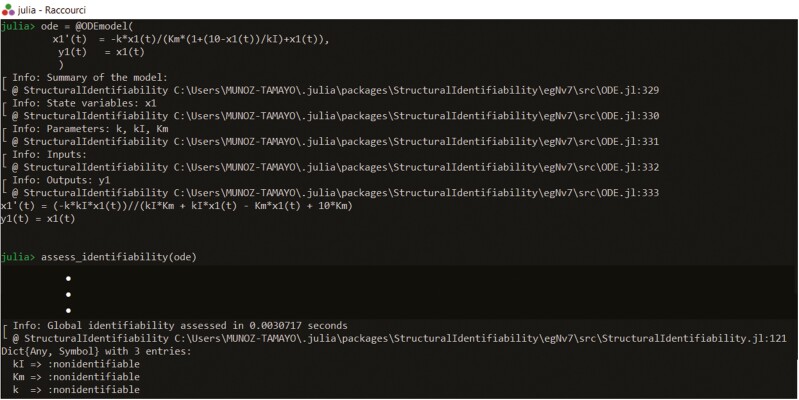
Output of identifiability testing of the model of β-casein hydrolysis with the StructuralIdentifiability software (Dong et al. 2022, preprint). The result of identifiability analysis is qualitative and gives information on which parameters are identifiable and which are not. In the example, all the parameters are non-identifiable.


Identifiability analysis was done with DAISY, NonlinearObservabilityTest, COMBOS, GenSSI 2.0, STRIKE-GOLDD 4.0, StructuralIdentifiability, and SIAN (implemented in Julia). The results led to the conclusion that the parameters $k,\;K_{m,\;\;\;}k_{I}$ are non-identifiable. Figure 2 shows the outcome of the analysis using StructuralIdentifiability. This result should not discourage us. We are actually in a very typical situation of over-parameterization (too many parameters). If we have information on prior values of any of the three parameters, we can set the parameter as known and let the other remaining parameters be estimated. Fixing the value of one parameter as known will render the other parameters globally identifiable. The previous solution directly reduces the number of parameters using prior knowledge of the parameter values. However, what happens if we do not have any prior information on any of the three parameters or if we set an incorrect prior value? A solution still exists; it is to re-parameterize the model. The model of this example is simple, and we can engage the parameterization by hand. By manipulation Eq. (5), we obtain the following reduced model, shown in Eqs (4)-(6)

$$\begin{array}{l} \frac{dx}{dt}=-b_{1}\cdot E\cdot \frac{x}{b_{2}-x} \end{array}$$ (6)

with


$$\begin{array}{l} b_{1}=\frac{k\cdot k_{I}}{K_{m}-k_{I}} \end{array}$$ (7)

$$\begin{array}{l} b_{2}=\frac{K_{m}\cdot \left(k_{I}+x_{0}\right)}{K_{m}-k_{I}} \end{array}$$ (8)

The parameters $b_{1},b_{2}$ are globally identifiable. The ­reparameterization helps here to improve the identifiability properties of the model. On the other hand, we lose parameter interpretability (Lema-Perez et al., 2019). The reparameterization task within the model-building process is indeed an exercise of trade-offs. In this simple example, the reparameterization can be done by inspection. However, for more complex models, the reparameterization can be challenging to reach by simple inspection. The Matlab application StrucID (Stigter and Joubert, 2021) allows the detection of the lack of identifiability in ODE models. The analysis provides information on correlations between potential non-identifiable parameters. This information can be further used within the procedure developed by Joubert et al. (2020) to obtain suitable reparameterizations to improve the identifiability of the model. The reparameterization process is, however, complicated and requires expert knowledge of mathematics and computer programming. In this regard, the COMBOS, STRIKE-GOLDD 4.0 tools, and the web application developed by Ilmer et al. (2021) provide helpful functionality since they allow the computation of identifiable combinations of parameters that are individually non-identifiable. These combinations can indeed inform reparameterizations for model simplification and to guarantee structural identifiability. Moreover, in some cases, the resulting identifiable combinations can have biological meaning for the system under study (Ilmer et al., 2021). The automatic reparameterization in STRIKE-GOLDD 4.0 is performed by the implementation of the AutoRepar procedure (Massonis et al., 2021). The web tool web application developed by Ilmer et al. (2021) uses the SIAN algorithm (Hong et al., 2019) for identifiability testing and the algorithm developed by Ovchinnikov et al. (2021) for computing identifiable combinations. GenSSI 2.0 has an implementation of parameter transformation to facilitate the removal of non-identifiable parameters.

## The Power of Selecting What to Measure

The following example uses a mathematical model describing methane production (CH_4_) by rumen methanogenic archaea in an in vitro batch system (Muñoz-Tamayo et al., 2019a). Equations (9) to (13) show the model equations.


$$\begin{array}{l} \frac{dx_{H_{2}}}{dt}=\mu_{max}\cdot exp\left(-\frac{K_{s}\cdot V_{g}}{n_{g,H_{2}}}\right)\cdot x_{H_{2}}-k_{d}\cdot x_{H_{2}} \end{array}$$ (9)

$$\begin{array}{lll} \frac{ds_{CO_{2}}}{dt}=\; & -\frac{-Y_{CO_{2}\cdot }\mu_{max}}{Y}\cdot exp\left(-\frac{K_{s}\cdot V_{g}}{n_{g,H_{2}}}\right)\cdot \; & x_{H_{2}}-k_{L}a\cdot \left(s_{CO_{2}}-K_{H,CO_{2}}\cdot R\cdot T\cdot n_{g,CO_{2}}/V_{g}\right) \end{array}$$ (10)

$$\begin{array}{l} \frac{dn_{g,H_{2}}}{dt}=-\frac{\mu_{max}}{Y}\cdot exp\left(-\frac{K_{s}\cdot V_{g}}{n_{g,H_{2}}}\right)\cdot V_{L}\cdot x_{H_{2}} \end{array}$$ (11)

$$\begin{array}{l} \frac{dn_{g,CO_{2}}}{dt}=V_{L}\cdot k_{L}a\cdot \left(s_{CO_{2}}-K_{H,CO_{2}}\cdot R\cdot T\cdot n_{g,CO_{2}}/V_{g}\right) \end{array}$$ (12)

$$\begin{array}{l} \frac{dn_{g,CH_{4}}}{dt}=\frac{Y_{CH_{4}\cdot }\mu_{max}}{Y}\cdot exp\left(-\frac{K_{s}\cdot V_{g}}{n_{g,H_{2}}}\right)\cdot V_{L}\cdot x_{H_{2}} \end{array}$$ (13)

where $s_{CO_{2}}\;$ is the concentration (mol/L) of carbon dioxide in the liquid phase, and $x_{H_{2}}\;$ is the biomass concentration (mol/L) of hydrogenotrophic methanogens. The number of moles in the gas phase is represented by the variables $n_{g,H_{2}},n_{g,CO_{2}},n_{g,CH_{4}}$. The constants $V_{g}$ and $V_{L}\;$ are the volume of the gas and liquid phase, respectively. Liquid-gas transfer for carbon dioxide is determined by the mass transfer coefficient $k_{L}a$ (h^−1^) and Henry’s law coefficient $K_{H,CO_{2}}\;$(M/bar). The constant R (bar⋅(M⋅K)^−1^) is the ideal gas law constant, and T is the temperature (K). The parameter $k_{d}$ (h^−1^) is the death cell rate constant. The parameters $Y,Y_{CO_{2}},Y_{CH_{4}}$ are the yield factors (mol/mol H_2_) of microbial biomass, CO_2_, and CH_4_ that account for the stoichiometry of the reactions. The model uses the microbial growth function proposed by Desmond-Le Quemener and Bouchez (2014), with hydrogen as the limiting substrate.


$$\begin{array}{l} \mu =\mu_{max}\cdot exp\left(-\frac{K_{s}\cdot V_{g}}{n_{g,H_{2}}}\right) \end{array}$$ (14)

where μ is the growth rate (h^−1^), $\mu_{max}$ (h^−1^) is the maximum specific growth rate constant and $K_{s}$(mol/L) the affinity constant. An implementation of the model in the Open Source software Scilab is available at https://doi.org/10.5281/zenodo.3271611.

Let us assume that only the concentration of methanogens $x_{H_{2}}$ and the moles of hydrogen $n_{g,H_{2}}$ are measured. We are interested in assessing the identifiability of the biological parameters $\mu_{max},\;K_{s,\;\;\;}k_{d},Y,Y_{CO_{2}},Y_{CH_{4}}$. All initial concentrations are known. The remaining (physical-related) parameters are known.

The previous model is non-rational since it includes an exponential function. Identifiability analysis was done with GenSSI 2.0 and STRIKE-GOLDD 4.0, which are a few tools that can analyze non-rational models. Under the ­observation conditions, the parameters $\mu_{max},\;K_{s,\;\;\;}k_{d},Y$ are globally identifiable, while $Y_{CO_{2}},Y_{CH_{4}}$are non-identifiable. The result of the non-identifiable parameters is not surprising. We can check by inspection of the model equations that it will be impossible to estimate the parameters $Y_{CO_{2}},Y_{CH_{4}}$ without measuring, respectively, CO2 and CH4. Indeed, we need the information on these quantities to estimate the relation of moles consumed or produced by mole of H2 utilized. Thus, the complete set of parameters is globally identifiable if $x_{H_{2},\;}n_{g,H_{2}},n_{g,CO_{2}},\;n_{g,CHO_{4}}$ are measured.

We can continue our analysis to illustrate the importance of integrating biological knowledge into the model. Methanogenesis is a process involving methane and microbial biomass production. We can represent the process in two reactions:


$$\begin{array}{l} R_{1}:4H_{2}+CO_{2}\to CH_{4}+2H_{2}O \end{array}$$

$$\begin{array}{l} R_{2}:10H_{2}+5CO_{2}+NH_{3}\to C_{5}H_{7}O_{2}N+8H_{2}O \end{array}$$

where C_5_H_7_O_2_N is the chemical formula for microbial biomass. Knowing the stoichiometry of the reactions enables us to reduce the number of yield parameters. The yield factor *Y* is the number of moles of microbial biomass produced per mole of H_2_ via reaction R_2_. We can then express the fraction (*f*) of H_2_ utilized in reaction R_1_ for methane production as a function of *Y*:


$$\begin{array}{l} f=1-10\cdot Y \end{array}$$ (15)

The number 10 in Equation (15) is the stoichiometry coefficient of H_2_ in R_2_. The yield factors of CO_2_ and CH_4_ can now be expressed as functions of *f*:


$$\begin{array}{l} Y_{CO_{2}}=\left(\frac{1}{4}\right)\cdot f+\left(\frac{5}{10}\right)\cdot (1-f) \end{array}$$ (16)

$$\begin{array}{l} Y_{CH_{4}}=\left(\frac{1}{4}\right)\cdot f \end{array}$$ (17)

This means that the number of parameters is now reduced to four parameters instead of 6. All the parameters are identifiable under the scenario where $x_{H_{2}}$ and $n_{g,H_{2}}$ are measured

## The Power of Analyzing Complex Models

**Figure 3.**
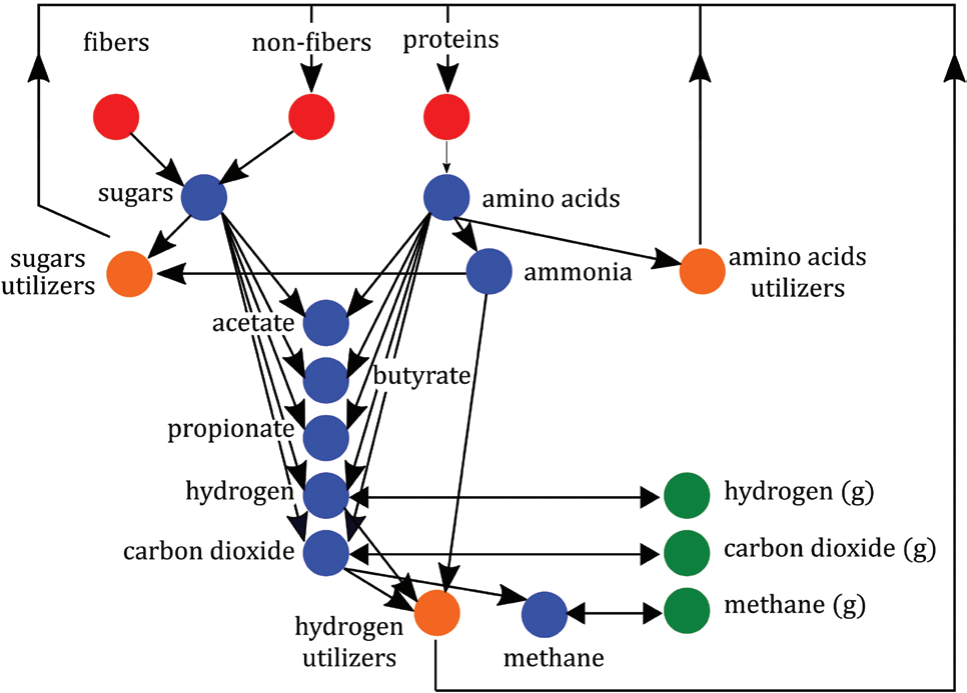
Schematics of the mathematical model of the rumen in vitro fermentation developed by Muñoz-Tamayo et al. (2016). Feed polymers (fiber, non-fiber carbohydrates, and proteins) are hydrolyzed into sugar and amino acid pools. The action of specific functional microbial groups further ferments these pools. Fermentation products are acetate, butyrate, propionate, carbon dioxide (CO_2_), and hydrogen (H_2_). In the liquid phase, the microbial group of hydrogen utilizers uses H_2_ and CO_2_ to produce methane (CH_4_). CO_2_, H_2_, and CH_4_ participate in a liquid-gas (g) transport phenomenon (represented by double arrows). Ammonia is used as the sole nitrogen source for hydrogen utilizers and sugar utilizers. Dead microbes are recycled in the trophic chain as non-fibers and protein polymers.

With the recent progress in computational methods, structural identifiability testing (at least locally) can be applied to complex nonlinear models. For example, previous studies (Ligon et al., 2018; Barreiro and Villaverde, 2022, preprint) showed that SIAN, StructuralIdentifiability, IdentifiabilityAnalysis, and GenSSI 2.0 were able to test the identifiability of models with more than 20 states variables and 20 parameters. IdentifiabilityAnalysis was used to assess the local structural identifiability of a model with about 100 states and 100 parameters (Karlsson et al., 2012). In the following example, we consider a mathematical model that represents the rumen fermentation under in vitro conditions (Muñoz-Tamayo et al., 2016). Figure 3 shows the schematics of the model. The model has 18 state variables and 30 parameters. A Matlab implementation of the model is freely available at https://doi.org/10.5281/zenodo.4047640. An implementation is also available in the R-package ­microPop (Kettle et al., 2018). An implementation in Scilab is also available at https://doi.org/10.5281/zenodo.4090332 for an extended model that accounts for the effect of the macroalgae *Asparagopsis taxiformis* on rumen fermentation and methane production (Muñoz-Tamayo et al., 2021). The original model includes algebraic equations to compute the pH dynamically. For our identifiability exercise, we set the pH to a constant value of 6.6.

We will consider the following 14 parameters for identifiability analysis: $k_{hyd,ndf}$ (hydrolysis rate constant of cell wall carbohydrates), $k_{hyd,nsc}$ (hydrolysis rate constant of non-structural carbohydrates), $k_{hyd,pro}$ (hydrolysis rate constant of proteins), $k_{m,su},$ (maximum specific utilization rate constant of amino sugars), $K_{s,su}$ (substrate affinity constant of sugars), $Y_{su}$ (microbial yield factor of sugars utilizers), $k_{m,aa}$ (maximum specific utilization rate constant of amino acids), $K_{s,aa,}$(substrate affinity constant of amino acids), $Y_{aa}$ (microbial yield factor of amino acids utilizers), $k_{m,H_{2}}$(maximum specific utilization rate constant of hydrogen), $K_{s,H_{2}}$ (substrate affinity constant of hydrogen utilization, $Y_{H_{2}}$ (microbial yield factor of hydrogen utilizers), and $\lambda_{1},\;\lambda_{2}$ (flux distribution parameters).

The initial conditions were set to be known. Identifiability testing was done with StructuralIdentifiability, GenSSI 2.0, and STRIKE-GOLDD 4.0. We run the tests on a laptop with Windows 64 Gb RAM, Intel Core i9-10885H (8 cores, 2.4 GHz). We first considered that 12 state variables were observed. The remaining unobserved state variables were the concentrations of CO_2_, H_2_, CH_4_ in the liquid phase and the concentration of the three microbial functional groups. The runtimes for the local structural identifiability analysis were 2.5 s for STRIKE-GOLDD 4.0, and 4.2 s for StructuralIdentifiability. The runtimes for global structural analysis were 2.6 min for StructuralIdentifiability and 25 min for GenSSI 2.0. Under the tested condition, the parameters are globally identifiable.

In the paper by Muñoz-Tamayo et al. (2016), the parameter estimation was defined for a subset of 10 parameters: $k_{hyd,nsc},\;k_{hyd,pro},\;k_{m,su},\;Y_{su},\;k_{m,aa},\;Y_{aa},\;k_{m,H_{2}},\;Y_{H_{2}},\lambda_{1},\;\lambda_{2}$. The remaining model parameters were fixed as known. This strategy was meant to facilitate the model calibration routine. The observed variables were the concentrations of acetate, butyrate, propionate, ammonia, and the moles of H_2_, CO_2_, and CH_4_ in the gas phase. When the model was built, no structural identifiability analysis was done. In the present exercise, we used StructuralIdentifiability and GenSSI 2.0 for identifiability testing. Both tools could not test the global identifiability of the parameters under the observation conditions. The analysis with both tools indicated that the model parameters are locally identifiable. The runtime with StructuralIdentifiability was 3.0 s. The runtime with GenSSI 2.0 was 34 min, but this time included the test on global identifiability, which was unsuccessful. STRIKE-GOLDD 4.0 exhibited an error in the process and could not assess the identifiability. We further evaluated the local structural identifiability of the 14 model parameters under the most restricted observation condition (that is, one single measurement). The result with StructuralIdentifiability informed that measuring any volatile fatty acid (acetate, butyrate, and propionate) yielded local identifiability of the parameters. This result is encouraging for the rumen modelers, although we might recognize that this outcome might appear surprising. The reason for the identifiability of the model parameters is associated with the nonlinear structure of the model. Although model complexity is not a reliable indicator to compare the identifiability properties between models (Roper et al., 2010), nonlinear complex models are likely more identifiable than linear models (Walter and Pronzato, 1996).

The identifiability results of the rumen fermentation model developed by Muñoz-Tamayo et al. (2016) are similar to those found by Nimmegeers et al. (2017) when studying the identifiability of the Anaerobic Digestion Model No.1 (ADM1). The ADM1 (Batstone et al., 2002) is a model developed to represent the digestion in reactors for wastewater treatment. The rumen fermentation model has a similar structure to that of ADM1, which explains the structural identifiability property shared between the models. For both models, a minimal set of measurements can guarantee local structural identifiability. Nimmegeers et al. (2017) explained that structural identifiability results from many interconnections between the model’s state variables. Such interconnection, however, also applies to the parameter set, leading to practical identifiability issues. Optimal experiment design can help to remediate practical identifiability issues, as discussed in the next section.

This example shows that existing identifiability analysis tools allow for handling complex models. Although for some models, it may not be possible to perform parameter identifiability analysis. What to do in these cases? We discuss some solutions here below.

### What can we do when identifiability testing is not possible?

Although developing advanced tools for structural identifiability testing might occur for some models with high complexity and limited observation conditions, current software tools cannot solve the identifiability problem. As mentioned above, from the tools shown in Table 1, only GenSSI 2.0 and STRIKE-GOLDD 4.0 can analyze non-rational models using symbolic computation. This can be a limitation issue in animal science, where non-rational functions (e.g., ­exponential ­functions) are common. Although, in some cases, ­transformations are possible to render the non-rational model in polynomial or rational form (see Muñoz-Tamayo et al., 2018 for an example of a transformation).

When identifiability tools fail to assess identifiability, the numerical approach implemented in the StrucID app can help identify correlations between potential non-identifiable parameters quickly. This valuable information can be used further to refine models and reduce the set of parameters to be checked for identifiability. The method implemented in StrucID app is not restricted to rational models. The methods developed within the practical identifiability framework (Lam et al., 2022; Villaverde et al., 2022) also provide valuable resources to assess a posteriori identifiability when structural identifiability testing is out of reach.

Finally, it is important to point out that automatic methods for identifiability testing are not free of error. In some cases, identifiability tools can yield incorrect results (Dong et al., 2022, Preprint). It is thus advisable to use different tools simultaneously to assess the correctness of the results (Joubert et al., 2021; Barreiro and Villaverde, 2022, preprint).

## The Power of Designing Optimal Experiments

Parameter identification is often addressed like a downstream process after collecting data. By following this approach, the modeler has minimal room for maneuvering to improve the model’s accuracy. The accuracy measures how closely the model predictions are to the actual values (Tedeschi, 2006). By incorporating identifiability analysis, we can follow an upstream approach to increase the room for maneuver of the modeler in the modeling construction process. The previous examples illustrate that the first benefit of identifiability analysis is providing valuable information about what to measure to render the model parameters identifiable. This part is done within the framework of structural identifiability. To complete the picture, we will need to know under which experimental conditions the measurements should be done on the real system to guarantee accurate parameter estimates. This part is addressed by practical identifiability analysis. To illustrate the usefulness of practical identifiability for optimal experiment design (**OED**) for parameter estimation, let us consider the following model that represents the utilization of a substrate $x_{2}$ by a microbe $x_{1}$ in a continuous system as follows:


$$\begin{array}{l} \frac{dx_{1}}{dt}=\frac{x_{1}\cdot x_{2}}{x_{2}+k+x_{2}^{2}/k_{I}}-D\cdot x_{1} \end{array}$$ (18)

$$\begin{array}{l} \frac{dx_{2}}{dt}=-\frac{x_{1}\cdot x_{2}}{x_{2}+k+x_{2}^{2}/k_{I}}+D\cdot (u-x_{2}) \end{array}$$ (19)

where D is the known dilution rate of the system, and u is the input substrate concentration. We would like to ­determine the shape of u to perform an experiment that allows estimating accurately the parameters $k,k_{I}$ from available measurements of $x_{1},x_{2}$. Under these observation conditions, the parameters are structurally globally identifiable. The accuracy of the estimates translates into small confidence intervals. Our objective function can be set up as an optimization problem where we want to find u such that the volume of the confidence intervals is minimized. One approach to address the OED problem is maximizing the determinant of the FIM. Muñoz-Tamayo et al. (2018) discussed the details of the calculation of the FIM and its use in the OED. This procedure requires defining nominal values for the parameters. For our example, we set $k=2,k_{I}=50$. We solve the OED problem for two cases. In the first one, we considered the input substrate concentration to be constant over time ($u_{c}$). In the second case, we considered the input substrate concentration to vary on time ($u_{d}$). For that we parameterized $u_{d}$ as a piecewise linear function.

The OED problem requires an intermediate level in computer programming skills. We used the IDEAS toolbox (Muñoz-Tamayo et al., 2009) to generate the functions for the OED problem. IDEAS is freely available at http://genome.jouy.inra.fr/logiciels/IDEAS. The files for the OED example are available at https://github.com/rafaelmunoztamayo/identifiability_examples. The toolbox Amigo 2 (Balsa-Canto et al., 2016) has the functionality of addressing OED problems for parameter estimation.

**Figure 4.**
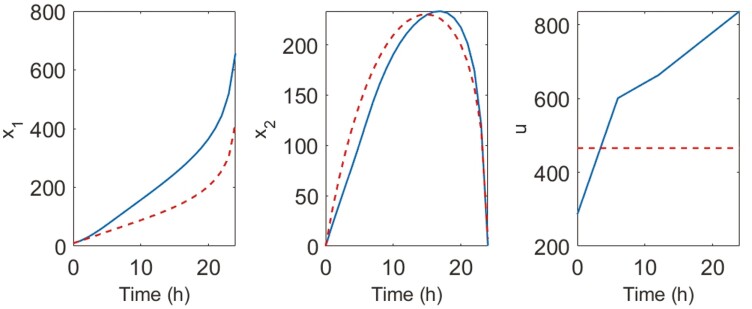
Responses of the variables of a mathematical model of microbial growth under two inputs of substrate concentration (*u*) were obtained to maximize the accuracy of the parameter estimates within an optimal experiment design. Dashed lines are the responses considering constant substrate input $u_{c}$ and solid lines are the responses for a dynamic substrate input $u_{d}$.

Figure 4 shows the obtained optimal inputs and the responses of the model variables. It is challenging to draw conclusions of this figure. However, when we look at the standard deviations (**SD**) of the parameter estimates obtained from the two cases in Table 2, we can clearly see the difference. The determinant of the FIM for $u_{d}\;$is 660 times higher than for $u_{c}$, which translates into smaller confidence intervals. The standard deviations obtained with $u_{d}$ are 31% and 72% those obtained with $u_{c}$ for k and $k_{I}$, respectively. Dynamic input induces better stimuli to system behavior and thus results in data with higher informative content than those obtained with constant input. This example shows the usefulness of OED in producing informative data for parameter estimation, and this capability can be used to avoid useless experiments. It should be said, however, that using the FIM for the calculation of confidence intervals is valid under linearity and asymptotic conditions (Walter and Pronzato, 1997). Approaches like the profile likelihood allow for overcoming the shortcomings of the FIM-based approach (Wieland et al., 2021).

**Table 2.** Comparison of the two optimal substrate inputs on the accuracy of the estimates of a model of microbial growth

	Constant input substrate $u_{c}$	Dynamic input substrate $u_{d}$
Determinant of the FIM	1.27 × 10^10^	8.40 × 10^12^
SD k	0.0247	0.0076
SD $k_{I}$	0.0282	0.0202

FIM, Fisher information matrix.

## Summary

**Figure 5.**
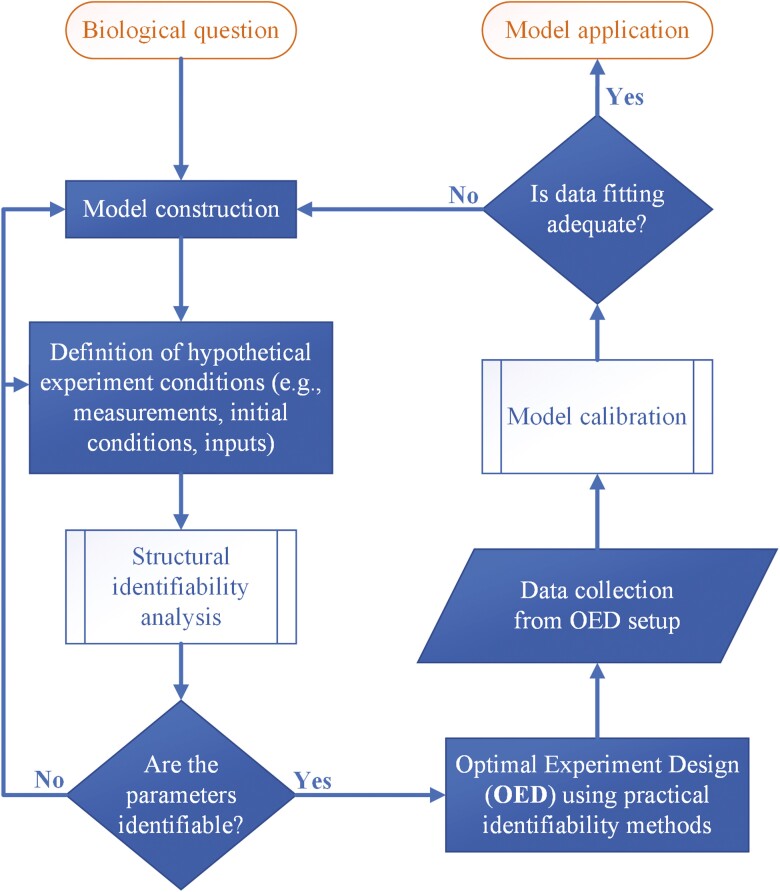
Workflow of the upstream approach proposed in this work. Structural and practical identifiability analysis and practical identifiability analysis are used before data collection to inform optimal experimental conditions. In the iterative process, experimental data and the mathematical model structure are modified to ensure that the model predictions are reliable.

In this paper, we showed that using an upstream approach that incorporates identifiability analysis before data collection in the workflow of model construction provides substantial benefits to obtaining reliable models. This workflow is displayed in Figure 5. Structural and practical identifiability analyses inform the conditions required to guarantee a unique and accurate estimation of the parameters. In case of lack of identifiability, identifiability analysis provides valuable information on possible actions to cure the non-identifiability (when possible). This information includes model reduction, reparameterization, and specifications on optimal measurements.

Existing freely available software tools enable the application of structural identifiability analysis without needing to be an expert in mathematics and computer programming. Recent software tools for structural identifiability analysis allow for handling complex models, but identifiability testing might be out of reach in some cases. In this case, numerical approaches within the practical identifiability framework can address the identifiability question. We believe that this paper will motivate the modeling community in animal science to integrate identifiability analysis in their model developments. Such integration can be easily done following a practitioner approach taking advantage of the variety of available software tools dedicated to identifiability testing. However, we must stress that the practitioner approach advocated in this paper is only possible thanks to the open science practices adopted by the parameter identifiability community in making their software toolboxes freely available. The parameter identifiability topic is an excellent example of how adopting open science practices can contribute to scientific progress. We, animal scientists, should learn from such efforts to make Open Science the new normal in our field (Muñoz-Tamayo et al., 2022). By sharing data, code scripts, and software tools and making our research freely accessible, we substantially strengthen the scientific progress of the animal science domain.

## Abbreviations

FIM: Fisher information Matrix
ODE: ordinary differential equations
OED: optimal experiment design
